# Answering the Call: Medical Students Reinforce Health System Frontlines Through Ochsner COVID-19 Hotline

**DOI:** 10.31486/toj.20.0065

**Published:** 2020

**Authors:** Justin J. Santos, Donald D. Chang, Katherine K. Robbins, Elise Le Cam, Anna Garbuzov, Monica Miyakawa-Liu, Bailey Goodwin, Iman Malik, Carl Tholen, Mark Green, Samantha Archie, Tiffany Tucker, Monique Ebberman, Gerald Dodd Denton

**Affiliations:** ^1^ The University of Queensland Faculty of Medicine, Ochsner Clinical School, New Orleans, LA; ^2^ Division of Academics, Ochsner Clinic Foundation, New Orleans, LA; ^3^ Patient Engagement and Outreach Division, Ochsner Clinic Foundation, New Orleans, LA; ^4^ Access to Care Division, Ochsner Clinic Foundation, New Orleans, LA; ^5^ Nursing Management and Clinical Operations, Ochsner Clinic Foundation, New Orleans, LA; ^6^ Department of Internal Medicine, Ochsner Clinic Foundation, New Orleans, LA

On Saturday evening, March 14, 2020, third- and fourth-year medical students at the Ochsner Clinical School (OCS) received an urgent email from their school administration:

Due to the growing COVID-19 pandemic, there have been over 1,500 calls to the Ochsner Call Line with over a 3-hour wait time…We are looking for volunteers to assist in answering the Ochsner Call Line and help triage patients.

The danger and scope of the pandemic were underscored the following day when the American Association of Medical Colleges ordered clinical rotations to pause.^1^

The idea for a student-run call center was born when the OCS administration, led by Leonardo Seoane, MD, FACP, Senior Vice President and Chief Academic Officer; G. Dodd Denton, MD, Assistant Dean and Associate Professor; and Carl Tholen, Assistant Vice President of Education Operations, were exploring options for how best to support patients, families, employees, and students amid the changes and safety restrictions implemented as COVID-19 emerged and began to spread in Louisiana. Anticipating the mandated pause in clinical rotations, the administrators recognized the value of engaging students in a support role. Clinical triage would provide both an educational opportunity for the medical students and an important resource for the community.

Within 24 hours after the initial call for volunteers was sent, OCS administration received responses from more than 100 students interested in staffing the call line. On Monday, March 16, 2 days after the call for volunteers, phones were installed at multiple stations in the academic testing center, and a crash course orientation was held in the afternoon. The following day, students began taking the first calls. From that point on, Ochsner medical students supported the nurse triage call line 12 hours per day (8:00 am to 8:00 pm), 7 days per week, from March 17 through April 26.

As with any project born out of a crisis, the call center required intense effort in the beginning. During the first week, 3 Ochsner student leads worked closely with call center staff each morning to ensure that the information provided to patients was up to date and to develop training materials for onboarding incoming volunteers. Samantha Archie from the Ochsner Access to Care Division was present in the call center Monday through Friday to provide technical support, ranging from call station functions to electronic medical record system (EMR) navigation. Monique Ebberman and Tiffany Tucker established medical student access to the EMR and managed EMR training and troubleshooting. Mark Green of the Patient Engagement and Outreach Division coordinated data aggregation for the call center and acted as liaison between the student leadership and Ochsner call center management. These members of the Ochsner call center team provided the necessary experience and expertise as the medical students familiarized themselves with the call center workflow.

One of the greatest challenges during the initial phase of operation was ensuring the students had the latest updates regarding available COVID-19 testing sites and specific screening criteria for each site. With the influx of information from multiple sources, including the Ochsner COVID-19 SharePoint website, NOLA Ready text updates, social media platforms, and the Louisiana Children's Medical Center (LCMC) COVID-19 Call Center, student leads and call center staff spent hours each day cross-referencing updates among sources and calling sites to verify testing criteria and availability status. The combined workload of continuously updating information and making sure volunteers of varying experience levels were trained and informed quickly overwhelmed the student leads. During the first week, the 3 leads worked both on shift, often in overlapping shifts, and at home as they designed training modules and workflow guides for volunteers. Training modules consisted of topics as diverse as how to work a phone to how to navigate the EMR. Workflow guides included a library of frequently asked questions; detailed scripts for fielding calls; and algorithms for determining screening recommendations for patients, as well as which testing site patients should be referred to based on patient location and site-specific criteria such as the presence and severity of symptoms, age and living conditions, and immunocompromised status.

At the end of the first week, the student leads created a shift coordinator system to distribute the workload. The coordinator roles were filled by 4 volunteers proficient with the call center workflow. Coordinators were tasked with training and briefing volunteers, assisting in updating information, and helping maintain continuity from shift to shift by performing handovers that included a review of any updates or challenging calls received during the prior shift. Any call that presented a unique problem was worth discussing, such as patients with atypical symptoms, calls from lesser-known areas of the state, or callers with special circumstances such as healthcare employees. The student coordinators developed a schedule to ensure that all 3 shifts were covered each day.

The impact of the student call center was quickly seen. By late March, 2 weeks after the first call was taken, the call line wait time had dropped from more than 3 hours to less than 10 minutes. During the first 3 weeks, more than 100 medical students staffed the call center, with 7 to 15 students taking calls every shift, volunteering nearly 2,000 hours, and assisting more than 5,200 callers. The most calls fielded in a single day was 409.

Assigning medical students to the Ochsner COVID-19 symptom tracking program was a natural extension of their call center role. The symptom tracking program was developed for patients who tested positive for COVID-19, had symptoms but did not meet criteria for testing, were awaiting test results, had received positive results and were still experiencing symptoms, and anyone else who may have had the virus and wanted to continue to be checked on. Beginning on March 29, the symptom tracking program provided an additional layer of support for patients to monitor their symptoms on a daily basis for 14 to 30 days and ensured they had a resource for their questions and assistance if they began to feel worse. Patients received daily text messages inquiring about their symptoms. Patients who responded with worsening symptoms or questions would be contacted by a nurse or medical student for triage. The goal was to make sure any patient who needed escalation of care was referred to the appropriate provider and to provide reassurance to patients during a time of uncertainty. Another goal was to ensure that patients understood their responsibility to prevent the virus from spreading by following the recommendations from the Centers for Disease Control and Prevention.

In early May, the medical students’ role continued to expand as they began to assist with the Ochsner COVID-19 prevalence study that involved testing more than 2,500 community members in Orleans and Jefferson parishes. The aims of this study were to gain an understanding of the spread of the virus, help healthcare providers and local leaders determine how many residents were infected, and to help inform how the city could safely reopen. Medical students answered community members’ questions related to the study, assisted people with study enrollment through the Test NOLA website, booked ride services for people without transportation, and directed patients to available testing sites.

Overall, the impact of the Ochsner medical students’ volunteerism extended beyond patient triage, symptom tracking, and study enrollment. Serving as a volunteer for the call center helped many medical students cope with the challenges of social distancing and the pause in their clinical training. Instead of staying home, the students served the community and safely engaged in meaningful human connection. They gained an understanding of and appreciation for healthcare roles that many had not previously encountered. Close collaborations with nurses, information technology staff, and administrators allowed the medical students to learn about their positions and create meaningful partnerships throughout the health system.

As Dr Denton pointed out, “We are living in a historical moment… and while medical students traditionally are restricted in what they can do, here is an opportunity where they can directly contribute and help stem the tide of the growing COVID-19 pandemic.”
